# Induction of Empathy by the Smell of Anxiety

**DOI:** 10.1371/journal.pone.0005987

**Published:** 2009-06-24

**Authors:** Alexander Prehn-Kristensen, Christian Wiesner, Til Ole Bergmann, Stephan Wolff, Olav Jansen, Hubertus Maximilian Mehdorn, Roman Ferstl, Bettina M. Pause

**Affiliations:** 1 Center of Integrative Psychiatry, University of Kiel, Kiel, Germany; 2 Department of Psychology, University of Kiel, Kiel, Germany; 3 Department of Neurology, University of Kiel, Kiel, Germany; 4 Department of Neuroradiology, Schleswig-Holstein University Hospital (UK-SH), Kiel, Germany; 5 Department of Neurosurgery, Schleswig-Holstein University Hospital (UK-SH), Kiel, Germany; 6 Department of Experimental Psychology, University of Duesseldorf, Duesseldorf, Germany; Victoria University of Wellington, New Zealand

## Abstract

The communication of stress/anxiety between conspecifics through chemosensory signals has been documented in many vertebrates and invertebrates. Here, we investigate how chemosensory anxiety signals conveyed by the sweat of humans (N = 49) awaiting an academic examination are processed by the human brain, as compared to chemosensory control signals obtained from the same sweat donors in a sport condition. The chemosensory stimuli were pooled according to the donation condition and administered to 28 participants (14 males) synchronously to breathing via an olfactometer. The stimuli were perceived with a low intensity and accordingly only about half of the odor presentations were detected by the participants. The fMRI results (event-related design) show that chemosensory anxiety signals activate brain areas involved in the processing of social emotional stimuli (fusiform gyrus), and in the regulation of empathic feelings (insula, precuneus, cingulate cortex). In addition, neuronal activity within attentional (thalamus, dorsomedial prefrontal cortex) and emotional (cerebellum, vermis) control systems were observed. The chemosensory perception of human anxiety seems to automatically recruit empathy-related resources. Even though the participants could not attentively differentiate the chemosensory stimuli, emotional contagion seems to be effectively mediated by the olfactory system.

## Introduction

Chemosensory alarm signals are supposed to have evolved independently within all major taxa, probably including plants [1] and are hypothesized to support evolutionary fitness [2]. In rodents, the release of chemosensory alarm signals is associated with activity of the pituitary-adrenal axis [3]. Different sensory systems are discussed to process stress-related social chemosignals in rodents (grueneberg ganglion cells [4], the vomeronasal organ [5], olfactory receptors [6], and trace-amine-associated receptors [7]). The chemosensory mediated alarm response in animals entails withdrawal behavior [8]–[10] and physiological adaptations [11], [12].

The processing of chemosensory anxiety signals affect perceptional performances by enhancing cognitive alertness [13], and reducing the perceptual acuity for social safety cues [14]. Furthermore, chemosensory stress signals of conspecifics augment defensive reflexes (startle) in humans [15] and rats [16]. However, the attentional capacities for the identification of chemosensory anxiety signals appear to be limited [17], [18].

The first brain imaging studies investigating the human brain response to social chemosignals have used single monomolecular substances contained in human body fluids [19], [20]. Hereby, brain regions involved in the coding of stimulus significance (amygdala, cingulate cortex) and in attentional control of stimulus processing (thalamus, parietal cortex) are activated. Additional hypothalamus activations seem to be related to inter-sexual communication of mating preferences [21]. Just recently, brain imaging studies have examined the brain's response to complex body odor signals. Hereby, it was shown that body odor in general is processed by brain structures outside the olfactory cortex (anterior and posterior cingulate cortex, occipital cortex [22]) and that smelling the body odor of significant others (body odors from strangers or relatives) activates brain structures involved in emotional and attentional stimulus processing, such as the insula and the precuneus [22], [23]. Another study, investigating the perception of the body odor of emotionally stressed odor donors (skydivers), focused on the amygdala's involvement in stress perception [24]. A fourth study found that the orbitofrontal and the fusiform cortex are activated during the perception of axillary sweat, sampled during a sexually arousing situation [25]. These activations have been discussed to be related to the social significance of the stimuli.

The present study aimed to investigate the neuronal correlates of the chemosensory perception of anxiety. Axillary sweat served as the anxiety signal and was collected from students while awaiting an oral examination at the university. The control sweat sample was obtained from the same participants while participating in an ergometer training.

## Materials and Methods

### Participants

Twenty-eight right-handed, non smoking undergraduate students (14 males) voluntarily participated in the experiment. All participants gave written informed consent and were paid for participation. None of them reported a history of chronic medication, of neurological, psychiatric, endocrine or immunological diseases, of diseases related to the upper respiratory tract, or skull injuries. None of the participants described themselves as being anxious in the magnetic resonance scanner (Magnet-Resonance-Fear Survey Schedule, [26]) and none of the participants experienced anxiety during the scanning procedure (State-Trait-Anxiety-Inventory, STAI-X1, [27]). The participants had a mean age of 22.1 years (SD = 2.9; range = 19–30 years), and males and females did not differ in age [t (26) = 0.65, p = 0.52]. The entire study, including the sweat sampling procedure, was conducted in accordance with the Declaration of Helsinki and was approved by the ethical committee of the medical faculty of the University of Kiel.

### Chemosensory stimuli

Axillary sweat was sampled by cotton pads over the course of one hour from 49 donors (28 males) in two situations: the first situation was a final oral examination at the university in order to acquire an academic degree (anxiety condition), and the second situation of sweat collection entailed a standardized ergometer training (sport condition).

The donors of the sweat samples were 24.3 years old (SD = 3.9, range = 20–37) and non-smokers. All of them reported to be of European origin, and not to be under acute or chronic medication. Furthermore, no participant indicated to suffer from any neurological, psychiatric, endocrine or immunological disease, or being involved in drug abuse. Their body-mass-index ranged between 18.3 and 28.8 (M = 22.6, SD = 2.4). The donors were instructed to refrain from eating garlic, onions, asparagus, or any other spicy food during the 24 hours prior to the odor donation. They were further advised to refrain from using deodorants within this timeframe, and to wash their armpits exclusively with an odorless medical soap (Eubos®, Dr. Holbein GmbH, Germany). All donors gave written and informed consent, and were paid for their donation.

In the anxiety condition, the cotton pads were fixed in donors' armpits 60 min before the oral examination started. At this time (baseline), 30 min before (t1), immediately before the examination started (t2), and subsequent to the 30 min examination (t3), saliva samples were collected to assess cortisol (Salivetten, Sarstedt AG & Co., Germany) and testosterone levels (SaliCaps, IBL, Germany). Immediately before the examination began, the cotton pads were removed and the donors described their current emotional state on the dimensions valence (happy–sad), arousal (aroused–relaxed), and dominance (dominant–submissive), using the Self Assessment Manikin (SAM, [28]). Additionally, they rated the intensity of six basic emotions (anxiety, joy, surprise, anger, sadness, disgust) on visual analogue scales.

The sport (control) condition consisted of three bicycling sets of 10 min duration each, where participants were requested to exercise at a constant heart rate of 110 bpm. The duration of the sport condition (60 min) equaled the waiting period prior to the examination (The introduction of the procedure lasted 10 min, and the ergometer training was separated by two breaks of 10 min each). A final 30 min resting period resembled the duration of the examination. In the beginning of the session, the cotton pads were fixed, and saliva samples were obtained at this point (baseline), after the first break (30 min later, t1), after the third bicycling set (60 min later, t2), and at the end of the session (90 min later, t3). Immediately after the third bicycling set (t2) the donors were asked to describe their current emotional state (SAM, basic emotions). Each donor participated in the two sessions on different days with each session being scheduled at the same hour of the day. On average, both sessions were scheduled 2.2 (SD = 0.6) days apart from each other.

Waiting for their oral examination, the donors experienced more anxiety [t (48) = 21.6, p<0.001] and less joy [t (48) = 9.0, p<0.001, see Table 1] as compared to the ergometer training. Even though all other basic emotions were experienced to a much lower degree, the donors felt more surprised [t (48) = 3.1, p<0.05], more angry [t (48) = 4.8, p<0.001], more sad [t(48) = 3.3, p<0.05], and more disgusted [t (48) = 3.0, p<0.05] during the anxiety condition than during the sport condition. In addition, donors reported feeling less happy and more submissive during the anxiety condition than during the sport condition [SAM: valence, t (48) = −9.14, p<0.001, SAM: dominance, t (48) = −7.21, p<0.001]. However, the arousal was experienced to be similar in both conditions [SAM: arousal t (48) = 1.87, p>0.20]. All t-test p-values were Bonferroni corrected.

**Table 1:** Emotions of the sweat donors (N = 49).

Rating	Dimension	Anxiety Condition		Sport Condition	
		M	SD	M	SD
**Basic Emotions**	**Anxiety**	6.15	1.9	0.36	0.54
	**Joy**	3.65	2.12	6.92	1.68
	**Surprise**	2.34	2.45	1.28	1.55
	**Anger**	1.8	2.04	0.49	0.59
	**Sadness**	1.6	1.85	0.66	1.08
	**Disgust**	1.13	1.7	0.48	0.81
**SAM**	**Valence**	0.06	1.41	2.49	1.32
	**Arousal**	6.29	1.38	5.86	1.35
	**Dominance**	4.63	1.20	6.45	1.29

Note: Basic Emotions: range 0–10; SAM: Valence: range −4–+4; Arousal: range 1–9, Dominance: range 1–9.

**Figure 1:**
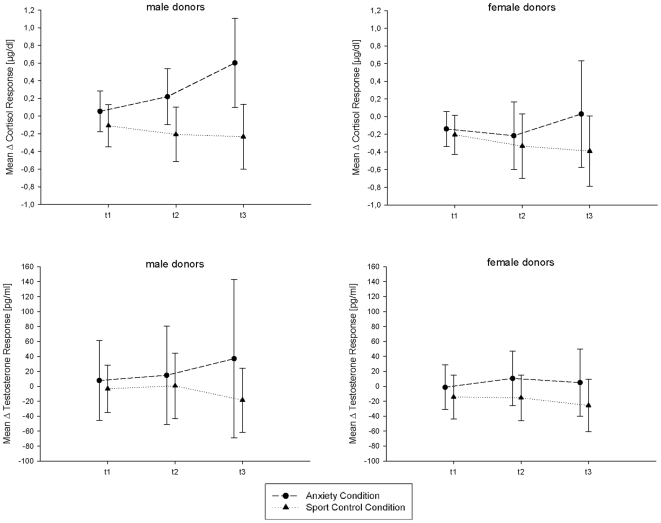
Cortisol (top) and testosterone (bottom) change-scores (difference values compared to the baseline measurement: means, SDs) for male (left) and female (right) donors at the three time points (t1, t2, and t3, separated by 30 min each).

The endocrine responses at all post-baseline periods were calculated with reference to the baseline. For each endocrine parameter an ANOVA with the factors Condition (anxiety, sport), Sex (male donor, female donor), and Time (t1, t2, and t3) was carried out. The cortisol level increased during the anxiety condition and decreased during the sport condition [Condition: F(1, 31) = 34.91, p<0.001; Condition×Time, F(2, 62) = 15.97, p<0.001]. In general, male donors showed a stronger cortisol increase than female donors [Time×Sex, F (2, 62) = 4.17, p<0.05]. Testosterone levels increased during the anxiety condition and decreased during the sport condition [Condition×Time, F(2, 60) = 5.30, p<0.05; see Figure 1].

Following the completion of collection, all sweat samples were pooled with distinction to the respective donation conditions and the donor's sex. Each of the four final homogenized samples were divided into small portions of 0.8 g (M = 0.805 g, SD = 0.001) and stored at −20°C. For the fMRI data recording, the small portions were filled into the glass bottles of the olfactometer and were renewed after each experiment.

### Olfactometer

According to Lorig and coworkers [29] a continuous airflow 6-channel olfactometer was constructed. Room air was pumped through a compressor into the system and passed a charcoal filter. A total air flow of 50 ml/s was divided into two independent currents: the carrier current (17 ml/s) and a second current (33 ml/s) which either passed an empty glass bottle during the interstimulus interval (ISI) or one of 4 odor bottles (male anxiety, male sport, female anxiety, and female sport). Whereas the carrier current was always active, computer controlled solenoid valves activated the second current. The switching valves in the control room were separated from the odor bottles, being placed near the scanner, by a 5 m long teflon tube. In order to prevent the odorized air from diffusing back down the tubing, the air flow passed a holdback (ca. 4 cm; main component polystyrene) after each glass bottle. Immediately before the air reached the participants, the low and the high currents converged to one current. The odors were delivered to the participants through a modified oxygen mask [30], which was connected to the odor bottles by a 2 m long teflon tube. Stimulus-onset latency after valve activation was about 0.9 s, and the stimulus rise-time was about 0.5 s (see supplementary material S1). The administration technique was validated by measuring the brain activation in response to a rose-like smelling odor (phenyl ethyl alcohol) in 8 participants (see supplementary material S1).

### Design and procedure

**Figure 2:**
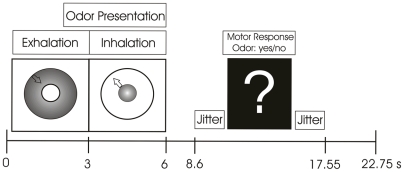
Trial time course. In the beginning of each trial, participants were requested to exhale (a ball decreased in diameter for 3 s) and then to inhale (a ball increased in diameter for 3 s). After a variable interval (range = 2.6–8.5 s) a question mark appeared on the screen and the participants were asked to indicate whether they perceived an odor or not. By pressing one of the two response buttons the questions mark disappeared. If no response was given within 2.75 s, an exclamation mark appeared for 0.5 s. The trial duration was 22.75 s.

During the fMRI scanning procedure (event-related design) each chemosensory stimulus (male anxiety, male sport, female anxiety, and female sport) was presented 20 times (pseudo randomized order). The stimuli were presented during four blocks (with 20 trials each), each block beginning with a dummy trial [31]. Visual instructions, presented by an MR-compatible monitor fixed at the sense coil, instructed the participants to inhale while the odors were delivered. Inhalation was preceded by an exhalation phase, during which subjects were presented with a ball on the monitor whose size decreased continuously across a period of three seconds. During inhalation the ball was presented with continuously increasing size, also for a period of three seconds (see Figure 2). To verify correct inhalation, a breathing belt was fixed around the chest at the site of the solar plexus. Online visual inspections of the breathing cycles revealed that all participants mastered correct breathing in more than 99% of all trials. Therefore, no data had to be excluded. On average 5.6 s (range = 2.6–8.5 s) after the end of the inhalation phase a question mark appeared, requesting participants to indicate whether they had perceived an odor or not. After pressing a response button the question mark disappeared. If no response was given within the next 2.8 s an exclamation mark occurred for 0.5 s. After a variable interval (mean duration: 8.1 s; range = 5.2–11.1 s) the next chemosensory stimulus was presented. The ISI and the total trial duration were fixed (ISI = 17.8 s; trial duration = 22.75 s). At the end of the session, the participants rated the degree of anxiety (STAI-X1, [27]) they experienced during the scanning procedure.

### fMRI data acquisition and analysis

Images were acquired using a 3 Tesla Intera Achieva (Phillips, NL) with a sense head-coil. A T1-weighted TFE-3D sequence was used for structural MRI of the whole brain [repetition time (TR) = 7.6 ms, echo time (TE) = 3.5 ms, flip-angle = 8°, 150 slices, slice thickness = 1 mm, gap: 0.1 mm, matrix: 224×224]. For functional imaging a single-shot T2*-weighted gradient echo-planar imaging sequence (EPI) was performed with 40 transversal slices covering the whole brain (TR = 3250 ms, TE = 35 ms, flip angle = 90°, slice thickness = 2.75 mm, gap: 0.25 mm, matrix: 80×80 voxels, in-plane resolution = 3×3 mm).

For the pre-processing and statistical analyses, the statistical parametric mapping software package (SPM5, Wellcome Department of Cognitive Neurology, London; www.fil.ion.ucl.ac.uk/spm) was used and implemented in Matlab (Mathworks, Inc., Natick, MA, USA release 14). Slice timing correction was performed and head motions across time were corrected by realigning and unwarping all scans to the first volume. Participants' T1-weighted images were co-registered to the corresponding mean EPI images and subsequently normalized to Montreal Neurological Institute standard space during the segmentation procedure, thus taking maximal advantage of the structural information in high-resolution T1-weighted images. EPI images were then normalized using the normalization-parameters written during segmentation of co-registered T1-weighted images [32] and spatially smoothed using an isotropic Gaussian kernel at 9-mm full width at half maximum.

For the individual subject analysis (first level), the conditions Male Anxiety Sweat, Female Anxiety Sweat, Male Sport Sweat, and Female Sport Sweat were specified as regressors. Furthermore, on basis of a single trial analysis, it was specified for each regressor whether or not the participants perceived the stimulus as an odor, resulting in 8 regressors in total. As the amount of perceived odors varied between the participants, the regressors were weighted in relation to the total number of trials for each participant and condition. At group-level (second level) the individual contrast images (collapsed over the conditions Smell/Non-Smell) were used in a flexible factorial design with Anxiety/Sport as within-subject factor and the between-subject factors Participant and Gender of Participant. In order to calculate simple effects of odor perception, a second flexible factorial design with the within-subject factor Odor Perception (collapsed over the conditions Anxiety/Sport) and the between-subject factors Participant and Gender of Participant was specified. Here, four subjects (three women) had to be excluded from the analysis, because they had either always or never detected the chemosensory stimuli as an odor. For the whole brain analyses the alpha error was set to 0.1%.

## Results

### Stimulus detection and ratings

Out of the chemosensory stimuli presented during scanning, the participants (N = 28, 14 males) detected on average 50.87% (SD = 22.27) as odors. Detection rates for the anxiety and sport odors were not significantly different [*F* (1, 26) = 2.74, p = 0.110].

Prior to the scanning session, the participants were asked to judge the intensity, pleasantness, unpleasantness and familiarity of the chemosensory stimuli (unipolar rating scales, range 0–8). The sweat samples were rated as low in intensity (M = 2.71, SD = 1.50), as weakly pleasant (M = 2.57, SD = 1.23) and also as weakly unpleasant (M = 2.48, SD = 1.71), and as low in familiarity (M = 2.43, SD = 1.51). The subjective ratings of the anxiety and sport odor were not significantly different [intensity: F (1, 26) = 0.08, pleasantness: F (1, 26 = 0.25, unpleasantness: F (1, 26) = 0.07, familiarity: F (1, 26) = 0.42; all p-values>0.50].

Additionally, participants were asked whether their feelings of happiness, arousal or dominance (SAM) were affected by one of the chemosensory stimuli. On average, participants did not report a significant change of emotions as a function of the different odors presented (all p-values>0.15).

### fMRI

#### Perception of chemosensory anxiety signals

**Figure 3:**
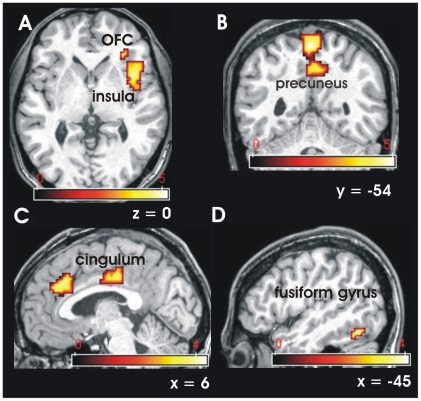
Brain activations of the contrast Anxiety minus Sport in 28 participants (threshold, p<0.001). A: Insula and OFC. B: Precuneus. C: Cingulate gyrus. D: Fusiform gyurs. OFC = orbitofrontal cortex.

**Figure 4:**
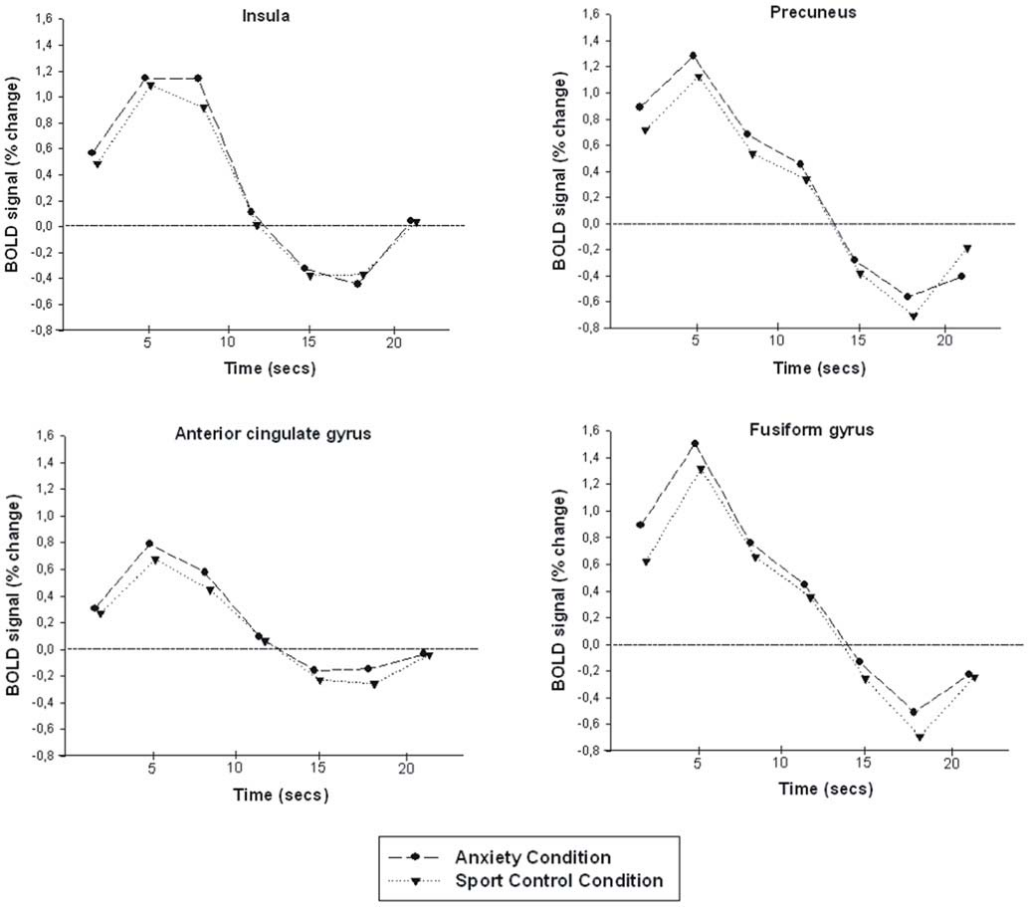
Time course of mean activations with respect to the anxiety and the sport control condition across all trials and participants [insula (x = 45 y = 12 z = 0), precuneus (x = 3 y = −54 z = 57), anterior cingulate gyrus (x = 6 y = 21 z = 21), fusiformis gyrus (x = −45 y = −60 z = −21)].

Contrasting the perception of anxiety sweat with sport sweat, significant brain activations were detected in the right insula (BA 44, 47, 48; Fig. 3a), the right precuneus (BA 4, 5; Fig. 3b), the left supramarginal gyrus (BA 40), the right thalamus, the dorsomedial frontal gyrus (BA 6, 8, 9), the right inferior frontal gyurs (BA 44), the right anterior (BA 24) and posterior (BA 23, 29) cingulated gyrus (Fig. 3c), the right substantia nigra, the left fusiform gyrus (BA 37; Fig. 3d), the left cerebellum (BA 19, 30) and the medial vermis (see Table 2). The hemodynamic response functions of the activations within the insula, the precuneus, the anterior cingulate gyrus and the fusiform gyrus are presented in Fig. 4. There were no significant activations contrasting Sport–Anxiety.

**Table 2:** Regional activations: Anxiety vs. Sport (N = 28).

Contrast	Activated Region		No. of Voxels	Z Score Primary Peak	MNI coordinates		
					X	Y	Z
**A>S**	Insula	R	263	4.62	45	12	0
				4.21	45	18	18
				3.94	54	12	18
	Insula/Inf. frontal gyrus, orbital part	R	32	4.47	33	30	−3
	Precuneus	R	494	4.50	3	−54	57
				4.26	−3	−36	72
				4.21	3	−42	69
	Supramarginal gyurs	L	41	3.75	−57	−54	30
				3.61	−60	−48	36
	Thalamus	R	150	4.43	9	−24	9
				3.69	6	−9	9
				3.57	18	−30	9
	Dorsomedial frontal gyrus	L/R	100	4.32	0	45	51
				3.72	−3	6	69
				3.41	3	21	63
	Inf. frontal gyrus, opercular part	R	60	4.17	48	15	36
				3.88	45	24	42
	Anterior cingulate gyrus	R	112	3.96	6	21	21
				3.76	0	30	27
				3.62	−3	27	36
	Posterior cingulate gyrus	R	113	3.82	3	−27	24
				3.8	0	−42	18
				3.68	0	−24	36
	Substantia nigra	R	52	3.92	6	−21	−21
	Fusiform gyrus	L	21	3.80	−45	−60	−21
	Cerebellum	L	54	3.72	−12	−42	−24
				3.39	−9	−33	−24
				3.47	−42	−69	−18
	Vermis	L/R	36	3.76	0	−54	−21
**S>A**	No activations						

Note: A = Anxiety Sweat; S = Sport Sweat; L = left; R = right; p<0.001; k>15.

#### Perception of the chemosensory stimuli as odors

Chemosensory stimuli which were detected as odors activated the right and left postcentral gyrus (BA 2, 3, 43), the right temporal gyrus (BA 37), the left thalamus, the left putamen (BA 48), and the right and left dorsomedial frontal gyrus (BA 46). The contrast between non-smelled stimuli and smelled stimuli revealed no significant brain activations (see Table 3).

**Table 3:** Regional Activations while perceiving an odor: Smelled stimuli vs. non-smelled stimuli.

Contrast	Activated Region		No. of Voxels	Z Score, Primary Peak	MNI coordinates		
					X	Y	Z
Smell>Non Smell	Postcentral gyrus	R	86	5.13	45	−33	60
				4.03	54	−30	51
	Postcentral gyrus	L	38	3.47	−57	−6	30
				3.38	−60	−3	21
	Medial temporal gyrus	R	81	4.47	51	−63	9
				3.87	51	−66	0
	Thalamus	L	86	4.08	−18	−12	18
				4.08	−15	−9	9
				3.73	−21	−24	6
	Putamen	L	17	3.96	−21	12	−9
	Dorsolateral frontal gyrus	L	21	3.64	−33	33	39
	Dorsolateral frontal gyrus	R	24	3.63	30	51	21
Non Smell>Smell	No activations						

Note: L = left; R = right; p<0.001; k>15.

## Discussion

Chemosensory signals of anxiety activate brain areas involved in the processing of social anxiety signals (fusiform gyrus), and structures which mediate the internal representation of the emotional state of others (insula, precuneus, cingulate cortex). In addition, the physiological adjustments to chemosensory anxiety signals include attentional control systems (dorsomedial prefrontal cortex, thalamus) and a supramodal unit, timing the different emotional processing systems (vermis, cerebellum). The chemosensory stimuli were judged to have a low intensity and only about half of the presentations were perceived as odors. The participants recognized the chemosensory stimuli of the anxiety and the sport-control condition as perceptually similar.

The perception of chemosensory anxiety signals most strongly activates the insula. Although insula activations are commonly observed during odor perception [33], in the present study these activations are very likely not caused by an olfactory component of the chemosensory anxiety signals. As the detection rates as well as the odor ratings did not differ between the two odors presented, it is rather likely that insula activations became induced by the social impact of the chemosensory anxiety signals. Comparing emotions evoked by social and non-social emotions has revealed that insula activity is specifically related to the decoding of social emotions [34] from facial and body signals [35]. It has been proposed that one major function of the insula in social communication is related to feelings of empathy [36]. In line with the potential role of the insular cortex to guide interoception [37], [38], the insula, in conjunction with the frontal operculum (which was also activated in the present study) might contribute to empathy by converting the feelings of others onto the internal body state of the perceiver [36].

A second major activation, associated with the perception of chemosensory anxiety signals, is located in the precuneus. The precuneus is strongly interconnected with the prefrontal cortex (BA 8, 9, 46), the premotor area, the supplementary motor area (SMA), and the anterior cingulate cortex [39]. This whole neuronal network was also activated through chemosensory anxiety signals. A key role of the precuneus seems to be related to self-referential stimulus processing. In detail, the precuneus seems to be involved in social communication by contributing to empathic judgements through distinguishing self from non-self perspectives [39], [40].

The activations of the anterior and posterior cingulate gyrus and the dorsomedial prefrontal cortex further support the assumption that the perception of chemosensory anxiety signals might release feelings of empathy. The cingulate gyrus is known to be activated during the processing of social information [34], [41], including body odors [22]. More specifically, the anterior and posterior cingulate cortex is involved in empathic, but not in non-empathic, mind-reading tasks [42]. Whereas the cingulate cortex might be responsible for the emotional perspective in empathy, the dorsomedial prefrontal cortex seems to be implicated in the attentional regulation of empathic feelings, regarding goal-directed behavioral adaptations [43]. Moreover, subcortical nuclei within the thalamus seem also to contribute to the attentional control systems, involved in the processing of chemosensory anxiety signals [44].

Activity within the fusiform cortex has been discussed as being selective for social (face) perception [45]. Concerning the results of the present study, it is most intriguing that the fusiform area responds most sensitive to social signals of anxiety [46], and that these social anxiety signals also include body expressions [47]. It is therefore postulated that the fusiform cortex plays a central role in the processing of social signals of anxiety, independent of the stimulus modality. Furthermore, the regulation of different emotional processing systems might also require the cerebellum, which might act as a pacemaker in maintaining the interaction between the processing systems at an optimum level. Especially the vermis has been considered to be involved in the regulation of negative mood states [48].

The exposure to chemosensory anxiety signals additionally activates the substantia nigra. Mesencephalic activations within the substantia nigra have been reported as being associated with higher order odor processing [49]. However, since the task requirements were equal for both stimuli, this interpretation of the results seems rather unlikely. As activity within the substantia nigra has recently been demonstrated as being related to novelty coding [50], [51], it could be speculated as to whether the anxiety signals comprised more uncommon and unexpected information than the chemosensory sport stimuli.

The here reported findings indicate a cluster of brain areas, responsible for chemosensory anxiety processing. In contrast, during the perception of axillary sweat sampled during an extreme stress situation (first-time tandem skydive), brain activity is more restricted to the amygdala [24]. Extreme physiological and psychological stress is not related to a specific emotion but activates a diverse set of physiological systems related to fight or flight behavior. It is therefore reasonable to assume that the perception of stress-related chemosignals does not activate emotion and empathy specific neuronal networks, but only less specific structures which effectively prime non-specific autonomic adjustments.

However, as only anxiety related signals were investigated in the present study, it can not be ruled out whether the here reported effects are solely related to the perception of anxiety. For the chemosensory modality, further studies are needed, separating the effects of different social emotions on central nervous systems.

In addition to the analysis of human brain activity which is associated with the perception of chemosensory anxiety signals, it has been examined whether the neuronal activity changes during the conscious perception of the chemosensory stimuli as odors. When the participants reported to smell an odor, neuronal activity was detected in thalamic dorsolateral frontal as well as in postcentral attentional control systems. The postcentral in conjunction with the dorsal frontal cortex seem to be a main relay station in the top-down control of attention [52], and the thalamus is considered to coordinate neocortical attentional control systems [44], thereby controlling the maintenance of attention [53]. Finally, the bilateral dorsolateral frontal activity might have been related to the involvement of working memory modules [54], coordinating attention and short-term-memory in order to detect the odors. It is concluded that reporting to smell an odor was caused by the recruitment of additional attentional resources.

In sum, the processing of chemosensory anxiety signals engages significantly more neuronal resources than the chemosensory processing of sport sweat. The odors were hardly detectable and the odors could not be differentiated regarding their intensity, pleasantness, unpleasantness or familiarity. Accordingly, it is concluded that the human brain automatically guides physiological adjustments to chemosensory anxiety signals, without being dependent on conscious mediation. However, in contrast to other modalities, the physiological adjustments in response to chemosensory anxiety signals seem to be mainly related to an automatic contagion of the feeling. In other words, smelling the feelings of others could be termed as an incorporation of the chemical expressions and thus the feelings of others.

## Supporting Information

Supplementary Material S1 Olfactometer
(0.06 MB DOC)
