# Supplementary material for: Induction of Empathy by the Smell of Anxiety
Source: PLoS One. 2009 Jun 24;4(6):e5987. doi: 10.1371/journal.pone.0005987 (PMC2695008; doi:10.1371/journal.pone.0005987)
Supplement: Supplementary Material S1 — Olfactometer (0.06 MB DOC) [file pone.0005987.s001.doc]

**Supplementary material**

**Induction of empathy by the smell of anxiety**

*Alexander Prehn-Kristensen^1^, Christian Wiesner^2^, Til Ole Bergmann^3^, Stephan Wolff^4^, Olav Jansen^4^, Hubertus Maximilian Mehdorn^5^, Roman Ferstl^2^ and Bettina M. Pause^6^*

^1^Center of Integrative Psychiatry, University of Kiel, 24105 Kiel, FRG

^2^Dept. of Psychology, University of Kiel, 24098 Kiel, FRG

^3^Department of Neurology, University of Kiel, 24105 Kiel, FRG

^4^Dept. of Neuroradiology, UK S-H, 24105 Kiel, FRG

^5^Dept. of Neurosurgery, UK S-H, 24105 Kiel, FRG

^6^Dept. of Experimental Psychology, University of Duesseldorf, 40225 Duesseldorf, FRG

Correspondence: Bettina M. Pause

Department of Experimental Psychology, University of Duesseldorf

Universitaetsstraße 1, D-40225 Duesseldorf

Tel.: +49 (0) 211 - 81-14384, Fax: +49 (0) 211 - 81-12019

E-mail: [bettina.pause@uni-duesseldorf.de](mailto:bettina.pause@uni-duesseldorf.de)

**Olfactometer**

*Stimulus onset latency and stimulus rise-time*

In order to measure the valve characteristics of the olfactometer, the odor glass bottles were filled with iced water (- 20° C) and the temperature deviations at the exit to the nose were measured with a thermistor. The duration of the valve activation was set to 3.5 s. An air flow of 33 ml/s either passed the iced bottle or an empty bottle during the ISI (duration = 3.5 s). Additionally, the carrier current was always active (17 ml/s). Fig. 1 shows the characteristics of a typical valve, averaged across 40 measurements. Stimulus onset latency is about 0.9 s, and the rise-time is about 0.5 s. Similarly, stimulus offset latency also takes about 0.9 s and it takes further 0.5 s until the temperature reaches the baseline level.

Fig. 1. Valve characteristics


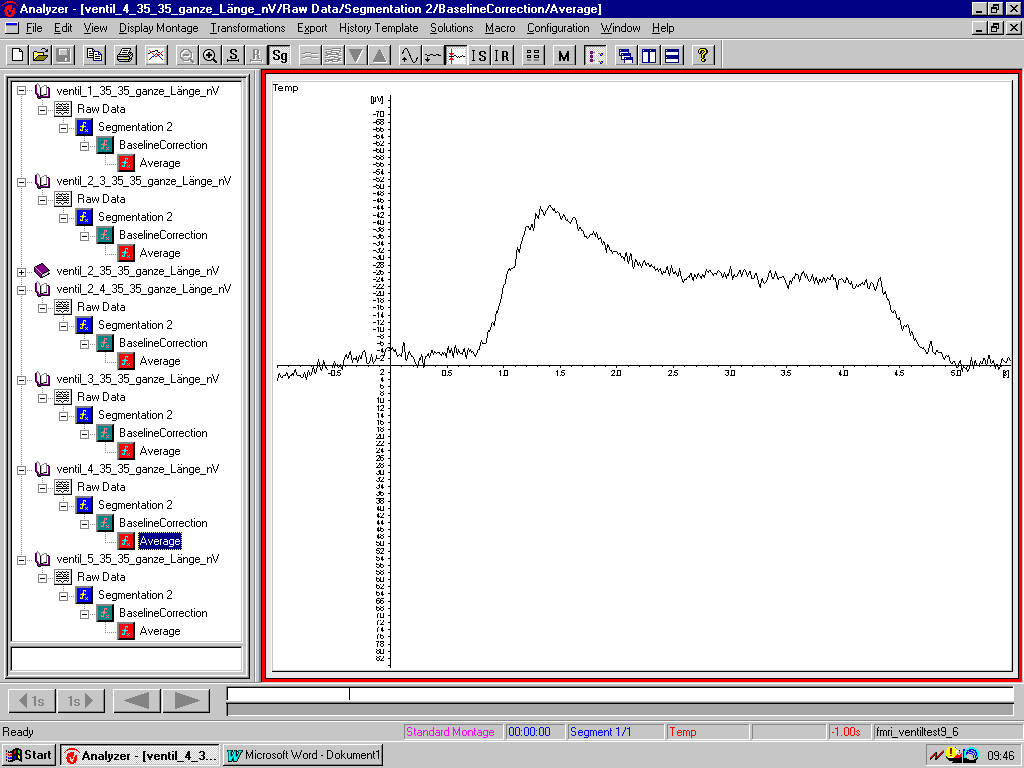


*Validation of the odor administration technique*

The aim of the validation study was to demonstrate activation in the olfactory cortex after the administration of an olfactory stimulus. Based on earlier fMRI studies of olfaction [1], three structures of the primary (piriform cortex, entorhinal cortex, and the amygdala) and two structures of the secondary olfactory cortex (hippocampus, orbitofrontal cortex) were defined as regions of interest (ROIs). The piriform cortex seems to have a number of integrative functions in odor processing, related to sniffing [2], selective attention [3], and long-term odor memory [4]. The amygdala and the orbitofrontal cortex are probably involved in the adjustment of motivational behavior during the perception of emotionally valenced odors [5]. The entorhinal cortex [6] and the hippocampus [7] seem to contribute to odor memory formation.

Eight healthy right-handed women (mean age: 27.0, SD = 6.3; range = 20-39) participated in this pre-study. A rose-like smelling olfactory stimulus (PEA, diluted in 1, 2-propanediol; 1:100 [v:v]; 1 ml odor dilution dropped on a cotton pad) and a control stimulus (pure cotton pad) were delivered by the olfactometer (for design and procedure see main study).

The fMRI session consisted of three runs, a total number of thirty trials per condition were included in the analysis. The individual data were pooled and a group-wise first level analysis was calculated (conditions: PEA, Control, Visual Countdown, Anticipation Response, and Response, see section fMRI data acquisition and analysi*s*). Thereafter, t-contrasts (PEA –Control) were calculated. The anatomical ROIs of the piriform cortex had to be determined manually, according to Mai et al. [8]. All other ROIs were detected according to the WFU-Pick-Atlas.

During the scanning procedure PEA was detected more often (98%) than the neutral control odor [24%; t (7) = 12.82, p < 0.001]. In addition, PEA was rated as being more intense [t (7) = 9.96, p < 0.001], more familiar [t (7) = 6.37, p < 0.001], and slightly more pleasant [t (7) = 2.07, p < 0.1] than the control stimulus.

Functional analysis (whole brain analysis; p = 0.001; uncorrected) revealed that PEA compared to the control stimulus activated the medial orbitofrontal cortex (MNI coordinates: x = 0, y = 60, z = -15; Z score = 6.46), the right entorhinal cortex/hippocampus (as a cluster within the gyrus temporalis inferior: MNI coordinates: x = 51, y = -6, z = -36; Z score = 4.71), the subcallosal gyrus [MNI coordinates (right): x = 6, y = 15, z = -12; Z score = 3.31; MNI coordinates (left): x = -3, y = 21, z = -12; Z score = 3.25], and the precuneus (MNI: coordinates x = 0, y = -51, z = -36; Z score = 4.95). Additional ROI analyses (p <0.05; uncorrected) showed that PEA compared to the control stimulus evoked stronger activities in the pirifom cortex (MNI coordinates: x = 24, y = -6, z = -12; Z score = 1.78) and the amygdala [MNI coordinates (right): x = 27, y = 3, z = 27; Z score= 1.78; MNI coordinates (left): x = -24, y =-3, z = -27; Z score = 1.84]. No ROI activation was detected in the contrast neutral control stimulus minus PEA.

**References**

1 Sobel N, Johnson BN, Mainland J, Yousem DM (2003) Functional neuroimaging of human olfaction. In: Doty RL, editor. Handbook of Olfaction and Gustation. New York: Marcel Dekker. pp. 251-273.

2 Sobel N, Prabhakaran V, Desmond JE, Glover GH, Goodes RL et al. (1998) Sniffing and smelling: separate subsystems in the human olfactory cortex. Nature 392: 282-286.

3 Zelano C, Bensafi M, Porter J, Mainland J, Johnson B et al. (2005) Attentional modulation in human primary olfactroy cortex. Nature Neurosci 8: 114-120.

4 Dade LA, Zatorre RJ, Jones-Gotman M. (2002) Olfactory learning: convergent findings from lesions and brain imaging studies in humans. Brain 125: 86-101.

5 Anderson AK, Christoff K, Stappen I, Panitz D, Ghahremani DG et al. (2003) Dissociated neural representations of intensity and valence in human olfaction. Nature Neurosci 6: 196-202.

6 Poellinger A, Thomas R, Lio P, Lee A, Makris N et al. (2001) Activation and habituation in olfaction – an fMRI study. NeuroImage 13: 547-560.

7 Savic I, Gulyas B, Larsson M, Roland P (2000) Olfactory functions are mediated by parallel and hierarchical processing. Neuron 26: 735-745.

8 Mai JK, Assheuer J, Paxinos G (2004) Atlas of the human brain. San Diego, CA: Elsevier Academic Press.
